# [Corrigendum] PRIMA-1 inhibits growth of breast cancer cells by re-activating mutant p53 protein

**DOI:** 10.3892/ijo.2026.5881

**Published:** 2026-04-06

**Authors:** Yayun Liang, Cynthia Besch-Williford, Salman M. Hyder

Int J Oncol 35: 1015-1023, 2009; DOI: 10.3892/ijo_00000416

Subsequently to the publication of the above article, an interested reader drew to the authors' attention that, regarding the immunohistochemical images shown in Fig. 1B on p. 1018, the 'PAb1620/PM 25μM' and 'PAb1620/PM 50μM' data panels shown in Fig. 1B for the T47-D and HCC-1428 cell lines were apparently the same, sugggesting that this figure had been assembled incorrectly.

After re-examining their original data, the authors have realized that the 'PAb1620/PM 25μM' and 'PAb1620/PM 50μM' data panels correctly shown for the T47-D cell line had inadvertently been copied across for the HCC-1428 cell line. The revised version of Fig. 1, now showing all the correct data for the HCC-1428 cell line in Fig. 1B, is shown below. The authors are grateful to the Editor of *International Journal of Oncology* for allowing them this opportunity to publish a Corrigendum, and all the authors agree to its publication. Note that this error did not grossly affect either the results or the conclusions reported in this study; furthermore, the authors apologize to the readership for any inconvenience caused.

## Figures and Tables

**Figure 1:**
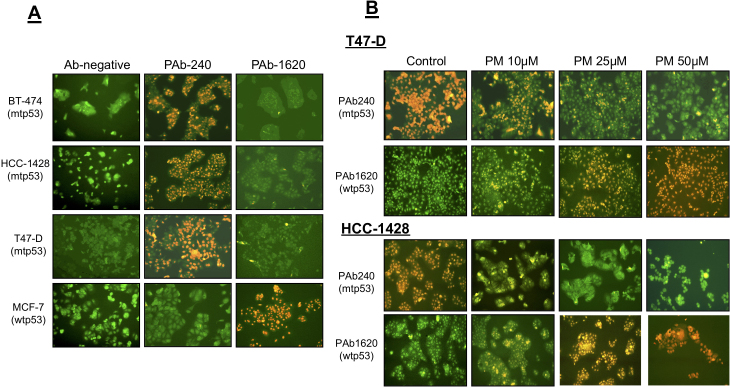
PRIMA-1 re-establishes the wt conformation of p53 in breast cancer cell lines expressing mtp53. (A) p53 status in BT-474, HCC-1428, T47-D, and MCF-7 cell lines as determined with the PAb 240 antibody, which recognizes only the mtp53 conformation and PAb 1620 which recognizes only wtp53 protein. Left panel represents controls where primary antibody was omitted, middle panel represents recognition of p53 with PAb240, and right panel represents recognition of p53 with PAb1620. (B) Cells were grown in 8-well chamber slides overnight and treated with PBS (control) or 10, 25, or 50 μM PRIMA-1 for 16 h. Treatment of breast cancer cells with PRIMA-1 converts the mtp53 protein (recognized by PAb240) to the wtp53 conformation, which is recognized by the conformation-specific antibody PAb1620. Results from T47-D and HCC-1428 cells are shown.

